# Deep Reinforcement Learning-Based Resource Allocation for UAV-GAP Downlink Cooperative NOMA in IIoT Systems

**DOI:** 10.3390/e27080811

**Published:** 2025-07-29

**Authors:** Yuanyan Huang, Jingjing Su, Xuan Lu, Shoulin Huang, Hongyan Zhu, Haiyong Zeng

**Affiliations:** 1 Guangxi Key Laboratory of Brain-Inspired Computing and Intelligent Chips, School of Electronic and Information Engineering, Guangxi Normal University, Guilin 541004, China; yyy_59o3o@163.com (Y.H.); sujingjing83@163.com (J.S.); luxuan2003611@163.com (X.L.); hsl5167@gxnu.edu.cn (S.H.); hyzhu-zju@foxmail.com (H.Z.); 2 Key Laboratory of Integrated Circuits and Microsystems, Education Department of Guangxi Zhuang Autonomous Region, Guangxi Normal University, Guilin 541004, China

**Keywords:** 3D trajectory design, deep reinforcement learning, resource allocation, non-orthogonal multiple access

## Abstract

This paper studies deep reinforcement learning (DRL)-based joint resource allocation and three-dimensional (3D) trajectory optimization for unmanned aerial vehicle (UAV)–ground access point (GAP) cooperative non-orthogonal multiple access (NOMA) communication in Industrial Internet of Things (IIoT) systems. Cooperative and non-cooperative users adopt different signal transmission strategies to meet diverse, task-oriented, quality-of-service requirements. Specifically, the DRL framework based on the Soft Actor–Critic algorithm is proposed to jointly optimize user scheduling, power allocation, and UAV trajectory in continuous action spaces. Closed-form power allocation and maximum weight bipartite matching are integrated to enable efficient user pairing and resource management. Simulation results show that the proposed scheme significantly enhances system performance in terms of throughput, spectral efficiency, and interference management, while enabling robustness against channel uncertainties in dynamic IIoT environments. The findings indicate that combining model-free reinforcement learning with conventional optimization provides a viable solution for adaptive resource management in dynamic UAV-GAP cooperative communication scenarios.

## 1. Introduction

The rapid development of the Industrial Internet of Things (IIoT) and smart manufacturing has imposed increasingly stringent requirements on wireless communication networks, particularly in industrial automation, predictive maintenance, and remote monitoring applications [1,2]. These applications demand massive device connectivity, ultra-high reliability, and extensive coverage, creating significant challenges for traditional terrestrial infrastructures. To address these challenges, unmanned aerial vehicles (UAVs) have emerged as a promising solution due to their high mobility, flexible deployment capabilities, and ability to dynamically adapt to communication demands. Serving as aerial base stations, UAVs can establish high-quality air-to-ground (A2G) line-of-sight (LoS) links [3,4], effectively complementing ground access points (GAPs) in complex industrial environments such as clean rooms, large factory sites, emergency scenarios, disaster recovery, and remote areas [5,6,7].

Early UAV-assisted communication systems primarily employed orthogonal multiple access (OMA) schemes for resource allocation, such as TDMA-based UAV scheduling [8] and hybrid cellular networks with UAVs as edge aerial base stations [7]. While OMA-based schemes ensure simple implementation and interference-free transmission, they often fall short in meeting the growing demands for spectral efficiency and massive connectivity in IIoT applications. To address these limitations, power-domain non-orthogonal multiple access (NOMA) has been introduced in UAV systems [9,10,11], enabling simultaneous multi-user transmission with improved spectral and energy efficiency. Studies have demonstrated that UAV-assisted NOMA provides superior performance than traditional TDMA schemes in terms of throughput, spectral efficiency, and coverage [12,13]. The recent literature has explored AI techniques in various NOMA paradigms, including Sparse Code Multiple Access (SCMA), which offers grant-free access capabilities for dense IIoT applications through optimized sparse codebook design [14,15]. AI algorithms have also proven effective in dynamic resource allocation for NOMA systems [16,17,18], with reinforcement learning being applied to optimize resource allocation and minimize Age of Information [19], demonstrating the versatility of AI techniques across different NOMA paradigms.

In UAV-assisted IIoT systems, the spectrum sharing between UAV and GAP introduces significant cross-tier interference, degrading communication performance. To mitigate this issue, cooperative transmission has been proposed to enable joint service by UAV and GAP [20]. Recent works have integrated cooperative transmission with NOMA and successive interference cancellation (SIC) techniques [21] and designed NOMA precoding matrices on the GAP side to effectively suppress interference while optimizing UAV trajectories [22]. However, most of the existing literature assumes exclusive user association with either UAV or GAP, limiting the exploitation of interference channels. A generalized joint user scheduling and power allocation (G-USPA) algorithm with 3D UAV trajectory design based on successive convex approximation (SCA) was proposed for UAV-assisted cooperative NOMA systems [7]. Though this approach demonstrated superior performance compared to non-cooperative systems, the SCA-based trajectory optimization incurs high computational complexity. Similarly, other related studies employing SCA methods and traditional convex optimization techniques [23,24] suffer from computational intensity and convergence challenges, limiting their adaptability in dynamic IIoT environments and making real-time deployment difficult [25,26]. Furthermore, UAVs in industrial environments are subject to limited battery capacity and computing power [27,28], necessitating more efficient and low-complexity resource management algorithms.

Deep reinforcement learning (DRL) has recently attracted significant attention for UAV decision-making and trajectory optimization in dynamic environments, thanks to its strong capabilities in feature extraction, policy learning, and adaptive decision-making under uncertainty. Unlike traditional optimization methods that require precise models and may struggle with environmental changes, DRL can learn flexible policies that adapt to varying conditions through interaction with the environment. Various DRL algorithms have been applied to this domain. For example, Deep Q-Networks (DQN) model the problem as a Markov Decision Process (MDP) to improve throughput and fairness [28], but their discretized action spaces limit precision. Continuous control methods such as Deep Deterministic Policy Gradient (DDPG) enhance accuracy in continuous domains [29], while Proximal Policy Optimization (PPO) incorporates clipped policy updates to enhance stability; it has also been successfully applied to large-scale network resource scheduling [30]. More recently, the Soft Actor–Critic (SAC) algorithm, leveraging a maximum entropy framework, offers improved convergence stability and efficiency, making it well suited for resource-constrained scenarios [31].

Based on the above analysis, there remains a need for efficient joint optimization approaches that can handle the complexity of UAV-GAP cooperative NOMA systems while ensuring real-time adaptability in dynamic IIoT environments. Unlike previous works that rely on traditional convex optimization solvers with high computational complexity [32] and poor real-time performance, this paper proposes a hybrid framework that combines traditional optimization techniques with deep reinforcement learning. This approach significantly reduces computational complexity compared to traditional convex programming methods while maintaining superior performance and real-time responsiveness suitable for complex industrial scenarios.

The main contributions of this work are summarized as follows:

- We propose a SAC-based joint optimization framework addressing UAV 3D trajectory planning and resource allocation challenges in dense and dynamic IIoT scenarios.
- By integrating power allocation, user scheduling, and 3D UAV trajectory design, we develop a joint resource management scheme for UAV-GAP cooperative NOMA systems that exploits interference channels to improve spectral efficiency and system throughput under stringent reliability and latency constraints.
- Simulation results validate the proposed approach’s performance improvements in IIoT downlink scenarios, including throughput gains, energy consumption reduction, and enhanced interference management, indicating its potential applicability in industrial contexts.

The remainder of this paper is organized as follows: Section 2 introduces the system model and problem formulation; Section 3 details the joint user scheduling and power allocation approach; Section 4 presents the trajectory optimization method based on DRL and SAC; Section 5 provides simulation results and discussions; Section 6 concludes this paper and discusses future research directions.

## 2. System Model and Problem Formulation

### 2.1. System Model

**Figure 1:**
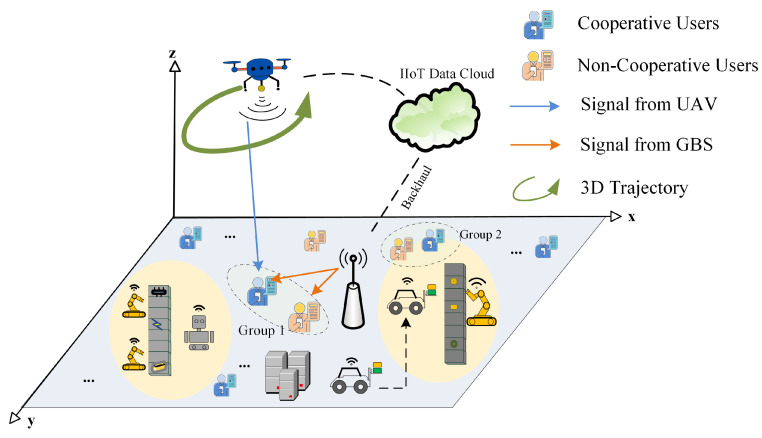
System model of UAV-GAP cooperative NOMA for IIoT, where all ground users are served by the GAP via NOMA and the UAV coordinates with the GAP to cooperatively transmit signals to cooperative users.

As illustrated in Figure 1, we consider a 3D downlink UAV-GAP cooperative communication system employing cooperative NOMA, serving a total of *K* terrestrial users. The UAV-BS flies above the coverage area and coordinates with the GAP to jointly serve these users. Users are divided into cooperative and non-cooperative groups based on their service modes, where different signal transmission strategies are employed to satisfy diverse, task-oriented, QoS requirements. Specifically, cooperative users are served jointly by both UAV and GAP to guarantee their QoS, while non-cooperative users are served only by the GAP. Both sets of users receive signals from the GAP under the NOMA scheme. We denote the sets of non-cooperative and cooperative users as $K_{c}$ and $K_{e}$, respectively.

We assume that the UAV flies periodically over the target coverage area with a cycle flight time *T*, which is divided into *N* equal time slots indexed by the set N={1,2,…,N}. The UAV’s 3D position at time slot *n* is denoted by $\left(x_{n},y_{n},z_{n}\right)$, where $z_{n}$ represents the UAV’s altitude. The horizontal coordinates of the GAP and the *i*-th terrestrial user are fixed at $\left(x_{b},y_{b},0\right)$ and $\left(x_{i},y_{i},0\right)$, respectively.

The channel state information (CSI) from the GAP to user *i* at time slot *n* is denoted by $h_{i,n}$, which is modeled as a Rayleigh fading channel with zero mean and unit variance. In contrast, the UAV-to-terrestrial user channel follows a probabilistic A2G line-of-sight/non-line-of-sight (LoS/NLoS) [33,34] propagation model. The Euclidean distance between the UAV and user *i* at time slot *n* is given by

(1) $$d_{i,n}=\sqrt{{\left(x_{n}-x_{i}\right)}^{2}+{\left(y_{n}-y_{i}\right)}^{2}+z_{n}^{2}}.$$

The corresponding elevation angle $\theta_{i,n}$ between the UAV and user *i* is

(2) $$\theta_{i,n}=\arccos\left(\frac{z_{n}}{d_{i,n}}\right).$$

The probability of LoS in the A2G channel between the UAV and user *i* is given by

(3) $$P_{\mathrm{LoS}}\left(\theta_{i,n}\right)=\frac{1}{1+ae^{-b(\theta_{i,n}-a)}},$$

where *a* and *b* are propagation constants that can be adjusted to different industrial environments [35,36,37]. The NLoS probability is $P_{\mathrm{NLoS}}\left(\theta_{i,n}\right)=1-P_{\mathrm{LoS}}\left(\theta_{i,n}\right)$.

Accordingly, the channel power gain between the UAV and user *i* at time slot *n* is expressed as

(4) $$v_{i,n}=\frac{\beta_{u}}{d_{i,n}^{\alpha }}P_{\mathrm{LoS}}\left(\theta_{i,n}\right)+\frac{\beta_{u}^{\prime }}{d_{i,n}^{\alpha^{\prime }}}P_{\mathrm{NLoS}}\left(\theta_{i,n}\right),$$

where $\beta_{u}$ and $\beta_{u}^{\prime }$ denote the channel power gains at the reference distance $d_{0}=1$ m under LoS and NLoS conditions, respectively, and α and $\alpha^{\prime }$ represent the corresponding path loss exponents.

As introduced above, the GAP adopts the NOMA strategy to serve all users. The users served cooperatively by the UAV and GAP are additionally served by the UAV via TDMA to guarantee their QoS requirements. Let $U_{n}$ denote the set of users served by the GAP at time slot *n* and denote the index of a cooperative user as *l*. Then, the received signal at an arbitrary user *k* served only by the GAP can be written as

(5) $$y_{k,n}=\sum_{k\in U_{n}}\left({\hat{h}}_{k,n}+e_{k,n}\right)\sqrt{P_{k,n}}S_{k,n}+v_{k,n}\sqrt{P_{u}}S_{l,n}+u_{k,n},$$

where $S_{k,n}$ and $S_{l,n}$ denote the transmitted symbols from the GAP to single-service user *k* and the UAV to cooperative user *l*, respectively, both with unit power, i.e., $|S_{k,n}{|}^{2}={\left|S_{l,n}\right|}^{2}=1$. $P_{k,n}$ and $P_{u}$ are transmit powers allocated by the GAP to user *k* and by the UAV, respectively. The noise term $u_{k,n}$ is additive white Gaussian noise with zero mean and variance $\sigma^{2}$, i.e., $u_{k,n}∼\mathrm{CN}\left(0,\sigma^{2}\right)$.

Define the true and estimated channel gains for user *k* at time slot *n* as $H_{k,n}={\left|h_{k,n}\right|}^{2}$ and ${\hat{H}}_{k,n}={\left|{\hat{h}}_{k,n}\right|}^{2}$, respectively. Given that ${\hat{h}}_{k,n}$ and $e_{k,n}$ are statistically independent, the expectation of the true channel gain satisfies

(6) $$E\left[H_{k,n}\right]=E\left[{\hat{H}}_{k,n}\right]+E\left[E_{k,n}\right],$$

where $E\left[E_{k,n}\right]=\sigma_{\mathrm{error}}^{2}$ represents the effect of imperfect CSI estimation.

At the receiver, SIC is performed to decode the composite signals [38]. For any user *k*, it sequentially decodes signals of users with higher decoding priorities before decoding its own signal by treating lower-priority users’ signals as noise [9]. To reduce SIC complexity and limit error propagation, we assume that each NOMA group contains exactly two users: one cooperative user and one user served solely by the GAP. Since the cooperative user typically experiences poorer channel conditions from the GAP due to longer distance or blockage, it is treated as the weak user relative to the other user in the same NOMA group.

For the user *k* served only by the GAP, the interference from the UAV’s signal must be accounted for. We denote the channel power gain from the UAV to this user as $V_{k,n}={\left|v_{k,n}\right|}^{2}$. Then, the signal-to-interference-plus-noise ratio (SINR) at user *k* after SIC is given by

(7) $$\gamma_{k,n}=\frac{{\hat{H}}_{k,n}P_{k,n}}{E_{k,n}P_{k,n}+V_{k,n}P_{u}+\sigma^{2}}.$$

The achievable data rate at user *k* in bits per second per Hz (bps/Hz) is

(8) $$R_{k,n}=\log_{2}\left(1+\gamma_{k,n}\right).$$

For the cooperative user *l*, which is served jointly by both UAV and GAP, the SINR after SIC can be expressed as

(9) $$\gamma_{l,n}=\frac{{\hat{H}}_{l,n}P_{l,n}+V_{l,n}P_{u}}{E_{l,n}P_{l,n}+\sigma^{2}+\left({\hat{H}}_{l,n}+E_{l,n}\right)P_{k,n}},$$

with the corresponding achievable rate

(10) $$R_{l,n}=\log_{2}\left(1+\gamma_{l,n}\right).$$

### 2.2. Problem Formulation

In this paper, we focus on jointly optimizing user scheduling, power allocation, and 3D UAV trajectory design to maximize the sum-rate of cooperative users while guaranteeing minimum rate requirements for all users to ensure fairness.

Let N={1,2,…,N} denote the set of time slots. We define the user scheduling indicator $C_{i,n}$, which equals 1 if user *i* is scheduled at time slot *n* and 0 otherwise. The sum-rate of cooperative users over all time slots is

$$R_{\mathrm{sum}}^{e}=\sum_{n\in N}\sum_{l\in K_{e}}C_{l,n}R_{l,n}.$$

Define the UAV position at time slot *n* as $\left(x_{n},y_{n},z_{n}\right)$. Its feasible 3D region is denoted by D, representing a bounded subset of the 3D space where the UAV is allowed to fly.

The optimization problem is formulated as

(11) $$\begin{array}{c} \mathrm{OP}1:\;\max_{\left\{C_{i,n},P_{i,n},\left(x_{n},y_{n},z_{n}\right)\right\}}R_{\mathrm{sum}}^{e} \end{array}$$

$$\begin{array}{cc} s.t.\;\; & \;\sum_{j\in U_{n}}P_{j,n}\leq P_{t},\;\forall n\in N;\left(11a\right)\\ \; & \;R_{k,n}\geq R_{\min},\;\forall n\in N,\forall k\in U_{n};\left(11b\right)\\ \; & \;\sum_{l\in K_{e}}C_{l,n}=1,\;\forall n\in N;\left(11c\right)\\ \; & \;\sum_{k\in K_{c}}C_{k,n}=1,\;\forall n\in N;\left(11d\right)\\ \; & \;\sum_{m=1}^{N}C_{k,m}\leq L_{c},\;\forall k\in K_{c};\left(11e\right)\\ \; & \;\sum_{m=1}^{N}C_{l,m}\leq L_{e},\;\forall l\in K_{e};\left(11f\right)\\ \; & \;\left(x_{n},y_{n},z_{n}\right)\in D,\;\forall n\in N;\left(11g\right)\\ \; & \;|x_{n+1}-x_{n}|\leq \frac{v_{\max,x}T}{N},\;\forall n=1,\ldots ,N-1;\left(11h\right)\\ \; & \;|y_{n+1}-y_{n}|\leq \frac{v_{\max,y}T}{N},\;\forall n=1,\ldots ,N-1;\left(11i\right)\\ \; & \;|z_{n+1}-z_{n}|\leq \frac{v_{\max,z}T}{N},\;\forall n=1,\ldots ,N-1.\left(11j\right) \end{array}$$

The constraints in problem **OP1** have the following interpretations. Constraint (11a) ensures that the total transmit power allocated by the GAP at each time slot does not exceed its maximum power budget $P_{t}$. Constraint (11b) guarantees that the achievable data rate of every scheduled user at each time slot meets or exceeds the minimum required rate $R_{\min}$, thus satisfying QoS requirements. Constraints (11c) and (11d) enforce that exactly one cooperative user and one non-cooperative user are scheduled in each time slot, ensuring fair and balanced resource allocation. Meanwhile, constraints (11e) and (11f) limit the total number of time slots that each non-cooperative user and cooperative user can be scheduled, respectively, through the upper bounds $L_{c}$ and $L_{e}$, preventing excessive scheduling of individual users. Constraint (11g) restricts the UAV’s position in each time slot to lie within the predefined 3D spatial region D. Lastly, constraints (11h) through (11j) impose velocity limits on the UAV’s movement along the *x*-, *y*-, and *z*-axes, respectively, by limiting the maximum displacement between consecutive time slots according to the UAV’s maximum allowable velocities $v_{\max,x}$, $v_{\max,y}$, and $v_{\max,z}$.

## 3. Joint User Scheduling and Power Allocation

### 3.1. Closed-Form Power Allocation with Given User Scheduling

Given a fixed user scheduling and UAV trajectory, the optimal power allocation for UAV-GBS CoMP-NOMA can be derived to maximize the sum-rate of CoMP users while ensuring minimum rate requirements for all users.

For each time slot *n*, consider the non-CoMP user $k_{1}$ and CoMP user $k_{2}$ scheduled, with their estimated channel gains satisfying ${\hat{H}}_{k_{1},n}\geq {\hat{H}}_{k_{2},n}$. Typically, due to poorer channel conditions, the CoMP user is regarded as the weak user in the NOMA pair, and the non-CoMP user as the strong one.

To determine the optimal power allocation, we analyze the relationship between the transmit power and rate of the non-CoMP user. According to the rate-to-power mapping, the transmit power allocated to user $k_{1}$ in time slot *n* is a function of its transmit rate $R_{k_{1},n}$. Differentiating $P_{k_{1},n}$ with respect to $R_{k_{1},n}$ yields

(12) $$\frac{\partial P_{k_{1},n}}{\partial R_{k_{1},n}}\geq 0,$$

which indicates that the transmit power $P_{k_{1},n}$ increases monotonically with $R_{k_{1},n}$.

Since a lower power allocation to the non-CoMP user reduces interference imposed on the CoMP user, it effectively increases the leftover power budget $P_{t}-P_{k_{1},n}$ available for the CoMP user to improve its achievable rate $R_{k_{2},n}$. Therefore, to maximize $R_{k_{2},n}$, the transmit power to non-CoMP user $k_{1}$ should be minimized, which corresponds to setting its transmit rate to the minimum required rate:

(13) $$R_{k_{1},n}^{*}=R_{\min}.$$

With this choice, the optimal transmit power allocated to the non-CoMP user is given by the closed-form expression:

(14) $$P_{k_{1},n}^{*}=\frac{\left(2^{R_{\min}}-1\right)\left(\sigma^{2}+V_{k_{1},n}P_{u}\right)}{{\hat{H}}_{k_{1},n}+E_{k_{1},n}}.$$

Consequently, the remaining power $P_{t}-P_{k_{1},n}^{*}$ is allocated to the CoMP user $k_{2}$, whose achievable rate can be expressed as

(15) $$R_{k_{2},n}^{*}=\log_{2}\left(1+\frac{{\hat{H}}_{k_{2},n}\left(P_{t}-P_{k_{1},n}^{*}\right)+V_{k_{2},n}P_{u}}{\sigma^{2}+E_{k_{2},n}\left(P_{t}-P_{k_{1},n}^{*}\right)+\left({\hat{H}}_{k_{2},n}+E_{k_{2},n}\right)P_{k_{1},n}^{*}}\right).$$

Although the power allocation problem is originally non-convex with respect to the power variables, it has been shown that expressing the sum transmit power as a convex function of users’ rates enables an equivalent convex optimization formulation over rates. This transformation facilitates efficient and tractable solution techniques.

In summary, given the fixed user scheduling and UAV trajectory, the above closed-form expressions yield an optimal power allocation strategy that satisfies minimum rate requirements and maximizes CoMP user rates, providing a foundation for joint optimization schemes.

### 3.2. Joint User Scheduling and Power Allocation Based on Bipartite Matching

In each time slot n∈N, the GAP jointly schedules one non-cooperative user $k_{1}\in K_{c}$ and one cooperative user $k_{2}\in K_{e}$ while simultaneously allocating transmit power to maximize system performance. To capture the coupling between user scheduling and power allocation, this problem is formulated as a maximum weight bipartite matching between the two disjoint sets of users at each time slot.

Specifically, we first compute the achievable rate of the cooperative user $k_{2}$ when paired with non-cooperative user $k_{1}$ in time slot *n* based on the closed-form power allocation derived in Section III-B. This achievable rate is denoted as $R_{k_{2},n}^{*}\left(k_{1},k_{2}\right)$. By evaluating all candidate user pairs, we construct a weight matrix $W_{n}\in R^{|K_{c}\left|\times \right|K_{e}|}$, whose entries are given by

(16) $$W_{n}\left(k_{1},k_{2}\right)=R_{k_{2},n}^{*}\left(k_{1},k_{2}\right).$$

The joint user scheduling and power allocation problem in time slot *n* thus reduces to selecting a matching $M_{n}$ between $K_{c}$ and $K_{e}$ that maximizes the sum of the corresponding achievable rates:

(17) $$\max_{M_{n}}\sum_{\left(k_{1},k_{2}\right)\in M_{n}}W_{n}\left(k_{1},k_{2}\right),$$

subject to the constraint that each user is scheduled at most once in the time slot, i.e.,

(18) $$|M_{n}\cap \left\{\left(k_{1},\cdot \right)\right\}|\leq 1,\;\forall k_{1}\in K_{c},\;\mathrm{and}\;\left|M_{n}\cap \left\{\left(\cdot ,k_{2}\right)\right\}\right|\leq 1,\;\forall k_{2}\in K_{e}.$$

This maximum weight bipartite matching problem can be efficiently solved by the Hungarian algorithm, which guarantees finding the optimal user pairing in polynomial time. Once the matching $M_{n}$ is obtained, the closed-form power allocation expressions are applied to each scheduled user pair to ensure that both transmit power constraints and minimum rate requirements are satisfied while maximizing the cooperative users’ achievable rates.

By independently applying this procedure to each time slot, the system performs efficient real-time joint optimization of user scheduling and power allocation that accounts for instantaneous channel conditions. The proposed framework achieves significant performance gains compared to heuristic and decoupled methods by integrating power allocation into the scheduling decision through the weight matrix. The maximum weight bipartite matching ensures optimal user pairing with polynomial-time complexity, making it suitable for real-time implementation. Furthermore, the framework provides flexibility to incorporate fairness constraints by modifying the weight matrix design. This approach offers a tractable solution for joint resource optimization in UAV-assisted cooperative NOMA systems.

## 4. Trajectory Optimization Using Deep Reinforcement Learning

### 4.1. Markov Decision Process Formulation

We model the UAV trajectory optimization as an MDP defined by the tuple (S,A,P,R,γ), capturing the interaction between the UAV agent and the wireless environment over discrete time slots indexed by *n*.

#### 4.1.1. State Space S

At time slot *n*, the state $S_{n}$ observed by the UAV agent includes its 3D position, time index, and instantaneous channel gains to all users:

(19) $$S_{n}=\left[x_{n},y_{n},z_{n},n,V_{1,n},V_{2,n},\ldots ,V_{U,n}\right],$$

where $\left(x_{n},y_{n},z_{n}\right)$ is the UAV location, *n* is the current time slot, and $V_{u,n}$ is the UAV-to-user *u* channel gain.

#### 4.1.2. Action Space A

The action $a_{n}=\left(v_{x,n},v_{y,n},v_{z,n}\right)$ represents the UAV’s velocity vector at time *n* with bounded components:

$$v_{x,n},v_{y,n}\in \left[-v_{\max},v_{\max}\right],\;v_{z,n}\in \left[-v_{\max,z},v_{\max,z}\right].$$

This continuous action space enables smooth 3D trajectory control.

#### 4.1.3. State Transition Probability P

The system state transitions according to the UAV’s mobility and wireless environment dynamics:

$$S_{n+1}∼P\left(S_{n+1}|S_{n},a_{n}\right).$$

Given the current position $\left(x_{n},y_{n},z_{n}\right)$ and velocity command $a_{n}$, the UAV position updates by

$$\left(x_{n+1},y_{n+1},z_{n+1}\right)=\mathrm{clip}\left(\left(x_{n},y_{n},z_{n}\right)+a_{n},D\right),$$

where clip(·) enforces spatial boundary constraints within the feasible flight domain D. The channel gains $h_{u,n+1}$ update as a function of the new UAV location and user positions following large-scale path loss models. The transition probability accounts implicitly for channel variations and system uncertainties. In our design, the transition model is unknown and learned implicitly by model-free reinforcement learning agents.

#### 4.1.4. Reward Function R

At each time slot *n*, the agent receives a scalar reward $r_{n}=R\left(S_{n},a_{n}\right)$, designed to simultaneously encourage high edge user rates, promote user fairness, and drive the UAV to return to its initial position. The reward is structured as follows:

(20) $$r_{n}=w_{1}R_{\mathrm{sum},n}^{e}+w_{2}r_{\mathrm{fair},n}+w_{3}r_{\mathrm{return},n}$$

where $R_{\mathrm{sum},n}^{e}=\sum_{l\in K_{e}}C_{l,n}R_{l,n}$ promotes maximizing edge user throughput, $r_{\mathrm{fair},n}$ penalizes unfair scheduling, and $r_{\mathrm{return},n}$ encourages the UAV’s return to its initial position. The weights $w_{1},w_{2},w_{3}$ are hyperparameters that control the relative importance of throughput maximization, fairness enforcement, and trajectory optimization, respectively.

#### 4.1.5. Discount Factor γ

The discount factor γ∈(0,1) trades off the immediate and future rewards, with larger values emphasizing long-term system performance and trajectory planning.

### 4.2. Soft Actor–Critic Algorithm

To solve the formulated MDP, we employ the SAC algorithm, which is particularly suitable for continuous control tasks such as UAV trajectory optimization. SAC is an off-policy, model-free deep reinforcement learning method that simultaneously maximizes expected cumulative reward and policy entropy, thereby fostering robust and sample-efficient learning in complex and high-dimensional environments.

SAC trains three parameterized functions concurrently: a stochastic policy (actor) $\pi_{\phi }\left(a|s\right)$ and two soft Q-functions (critics) $Q_{\theta_{1}}\left(s,a\right)$ and $Q_{\theta_{2}}\left(s,a\right)$, parameterized by ϕ, $\theta_{1}$, and $\theta_{2}$, respectively. The actor outputs a probability distribution over continuous actions, enabling effective exploration, while the dual critics reduce overestimation bias through clipped double Q-learning.

**Figure 2:**
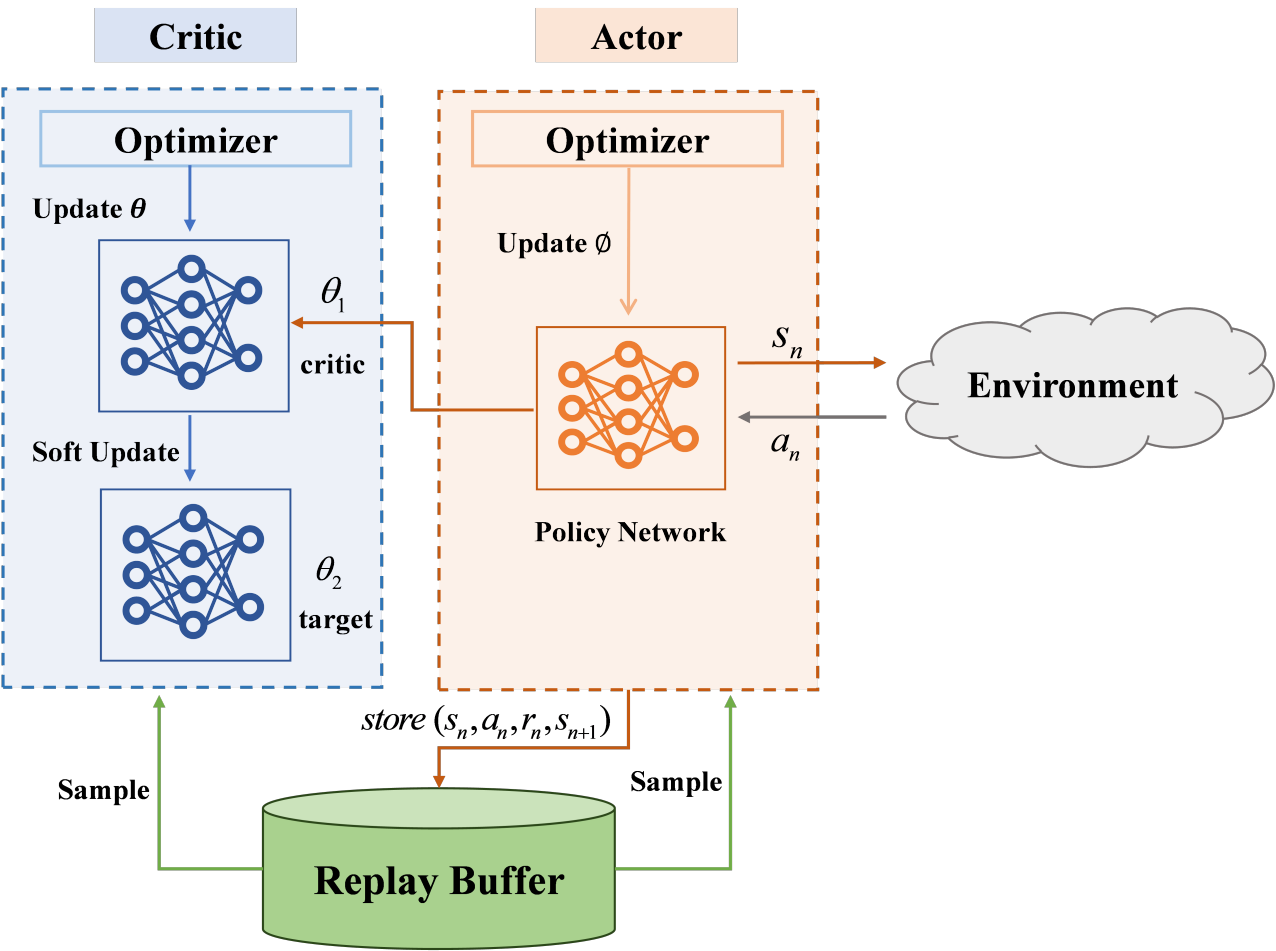
Soft Actor–Critic framework for UAV trajectory and joint scheduling optimization in a UAV-GAP cooperative NOMA system.

The overall architecture of the Soft Actor–Critic algorithm for the UAV-GAP cooperative NOMA system is illustrated in Figure 2. This framework integrates the UAV’s continuous trajectory control, represented as velocity commands, with the joint user scheduling and power allocation coordinated between the UAV and the ground base station. The SAC agent observes the current system state, including UAV position and channel conditions, and outputs continuous control actions to optimize long-term performance.

Contrary to conventional RL algorithms that optimize expected return alone, SAC maximizes a maximum entropy objective, defined as

(21) $$J\left(\pi \right)=\sum_{n}E_{\left(s_{n},a_{n}\right)∼\rho_{\pi }}\left[r\left(s_{n},a_{n}\right)+\alpha H\left(\pi (\cdot |s_{n})\right)\right],$$

where $H(\pi \left(\cdot |s_{n}\right))$ represents the Shannon entropy of the policy at state $s_{n}$ and α>0 is the temperature parameter governing the trade-off between exploration and exploitation. This entropy regularization promotes stochasticity in the policy, encouraging diverse behaviors and avoiding premature convergence to suboptimal deterministic policies.

For stable training, SAC utilizes two Q-functions approximated by neural networks and employs clipped double Q-learning to mitigate positive bias in value estimates. Experience replay buffers and target networks further enhance sample efficiency and convergence stability.

During training, each critic network minimizes the soft Bellman residual:

(22) $$L\left(\theta_{i}\right)=E_{(s_{n},a_{n},r_{n},s_{n+1})∼D}\left[{\left(Q_{\theta_{i}}\left(s_{n},a_{n}\right)-y_{n}\right)}^{2}\right],\;i\in \left\{1,2\right\},$$

where the target $y_{n}$ is computed by

(23) $$y_{n}=r_{n}+\gamma E_{a_{n+1}∼\pi_{\phi }}\left[\min_{j=1,2}Q_{\theta_{j}^{\prime }}\left(s_{n+1},a_{n+1}\right)-\alpha \log\pi_{\phi }\left(a_{n+1}|s_{n+1}\right)\right],$$

and $\theta_{j}^{\prime }$ denotes the parameters of the slowly updated target critic networks.

The actor updates its policy by minimizing the expected Kullback–Leibler divergence between $\pi_{\phi }$ and the exponential of the Q-function, effectively maximizing

(24) $$J\left(\phi \right)=E_{s_{n}∼D,a_{n}∼\pi_{\phi }}\left[\alpha \log\pi_{\phi }\left(a_{n}|s_{n}\right)-\min_{j=1,2}Q_{\theta_{j}}\left(s_{n},a_{n}\right)\right].$$

The temperature parameter α can be adjusted automatically to maintain a target entropy, further improving exploration and training stability.

In the context of UAV cooperative NOMA systems, SAC’s capabilities to handle continuous 3D velocity actions and noisy, high-dimensional observations such as UAV position and channel gains are crucial. The entropy-regularized policy encourages diverse trajectory exploration, which helps the UAV avoid suboptimal flight patterns caused by the highly non-convex wireless environment and dynamic user scheduling.

Overall, SAC combines rigorous theoretical foundations with practical efficacy, making it an ideal approach for the UAV trajectory optimization problem. The detailed procedure of the Soft Actor–Critic-based trajectory optimization is summarized in Algorithm 1, which outlines the main steps of network initialization, interaction, training updates, and policy improvement.
**Algorithm 1** Soft Actor–Critic-Based Trajectory Optimization  1:Initialize actor network $\pi_{\phi }\left(a|s\right)$ and two critic networks $Q_{\theta_{1}}\left(s,a\right)$, $Q_{\theta_{2}}\left(s,a\right)$ with random weights $\phi ,\theta_{1},\theta_{2}$.  2:Initialize target critic networks with weights $\theta_{1}^{\prime }\leftarrow \theta_{1},\;\theta_{2}^{\prime }\leftarrow \theta_{2}$.  3:Initialize temperature parameter α and replay buffer D.  4:**for** episode = 1 to MaxEpisodes **do**  5:      Reset environment and obtain initial state $s_{0}$.  6:      **for** time step n=0 to N−1 **do**  7:            Sample action $a_{n}∼\pi_{\phi }\left(\cdot |s_{n}\right)$.  8:            Execute $a_{n}$; observe reward $r_{n}$ and next state $s_{n+1}$.  9:            Store $\left(s_{n},a_{n},r_{n},s_{n+1}\right)$ in D.10:            Sample random minibatch of size *M* from D.11:            **for** i=1 to *M* **do**12:                 Compute target value:$$y_{i}=r_{i}+\gamma E_{a_{i+1}∼\pi_{\phi }}\left[\min_{j=1,2}Q_{\theta_{j}^{\prime }}\left(s_{i+1},a_{i+1}\right)-\alpha \log\pi_{\phi }\left(a_{i+1}|s_{i+1}\right)\right].$$13:            **end for**14:            Update critics $\theta_{1},\theta_{2}$ by minimizing:$$L\left(\theta_{j}\right)=\frac{1}{M}\sum_{i=1}^{M}{\left(Q_{\theta_{j}}\left(s_{i},a_{i}\right)-y_{i}\right)}^{2},\;j=1,2.$$15:            Update actor ϕ by minimizing:$$J\left(\phi \right)=\frac{1}{M}\sum_{i=1}^{M}\left[\alpha \log\pi_{\phi }\left(a_{i}|s_{i}\right)-\min_{j=1,2}Q_{\theta_{j}}\left(s_{i},a_{i}\right)\right].$$16:            (Optional) Adjust temperature α to control entropy.17:            Soft update target networks:$$\theta_{j}^{\prime }\leftarrow \tau \theta_{j}+\left(1-\tau \right)\theta_{j}^{\prime },\;j=1,2;\;\phi^{\prime }\leftarrow \tau \phi +\left(1-\tau \right)\phi^{\prime }.$$18:      **end for**19:**end for**20:**return** Learned policy $\pi_{\phi }\left(a|s\right)$ and optimized UAV trajectory.

### 4.3. Computational Complexity Analysis

This section analyzes the computational complexity of the proposed joint optimization framework to demonstrate its practical feasibility for real-time IIoT applications.

The complexity analysis considers our hybrid optimization design, which enables distributed implementation. In this design, resource allocation computations occur at the ground access point, while trajectory control runs on the UAV. The SAC policy network requires minimal computational resources for online execution through a single forward pass. The discrete time slot operation provides adequate time for optimization between transmission periods.

#### 4.3.1. Joint User Scheduling and Power Allocation

The resource allocation algorithm operates in two sequential phases for each time slot. First, the algorithm computes the weight matrix by evaluating achievable rates for all possible user pairs. Given the closed-form power allocation solutions derived in Section 4.1, the rate computation for each cellular-edge user pair $\left(k_{1},k_{2}\right)$ requires constant time operations. Therefore, constructing the complete weight matrix has complexity $O(|K_{c}|\cdot |K_{e}|)$.

Second, the maximum weight bipartite matching problem is solved using the Hungarian algorithm. For a bipartite graph with $|K_{c}|$ cellular users and $|K_{e}|$ edge users, the Hungarian algorithm requires $O((|K_{c}\left|+\right|K_{e}{\left|\right)}^{3})$ operations. Since the matching complexity typically dominates the weight computation for practical system sizes, the overall per-time-slot complexity is

(25) $$O\left(\max\{|K_{c}\left|\cdot \right|K_{e}\left|,(\right|K_{c}\left|+\right|K_{e}{\left|\right)}^{3}\}\right)$$

#### 4.3.2. SAC-Based Trajectory Optimization

The SAC algorithm involves distinct training and inference phases with different computational requirements.

(1) Training Phase: The SAC framework maintains one actor network and two critic networks, along with corresponding target networks. During each training step, the algorithm performs forward and backward passes through all networks. With *E* training episodes, *N* time slots per episode, and neural networks having *L* layers with *H* hidden units each, the training complexity is

(26) $$O\left(E\cdot N\cdot \left(\underset{\mathrm{Two}\;\mathrm{Critics}}{\underset{︸}{2LH^{2}}}+\underset{\mathrm{Actor}}{\underset{︸}{LH^{2}}}+\underset{\mathrm{Target}\;\mathrm{Updates}}{\underset{︸}{LH^{2}}}\right)\right)=O\left(4E\cdot N\cdot L\cdot H^{2}\right)$$

(2) Inference Phase: During online operation, only the trained actor network executes policy decisions, requiring $O(L\cdot H^{2})$ operations per time slot.

#### 4.3.3. Overall System Complexity

The total online computational complexity per time slot combines both resource allocation and trajectory optimization components:

(27) $$O\left(\max\{(|K_{c}\left|+\right|K_{e}{\left|\right)}^{3},L\cdot H^{2}\}\right)$$

The polynomial-time complexity ensures computational tractability for practical IIoT deployments. For typical deployment scenarios with moderate numbers of users and compact neural network architectures, the computational overhead remains within the processing capabilities of modern edge computing platforms, enabling real-time system operation.

## 5. Results and Discussion

In this section, we evaluate the performance of the proposed joint user scheduling and power allocation scheme in the UAV cooperative NOMA system through extensive simulations. The simulation settings and key system parameters [32] are summarized in Table 1.

For the SAC-based trajectory optimization, we employed a deep neural network architecture with appropriate hyperparameter settings to ensure stable learning and optimal convergence performance. The specific training parameters for the SAC algorithm are detailed in Table 2.

**Figure 3:**
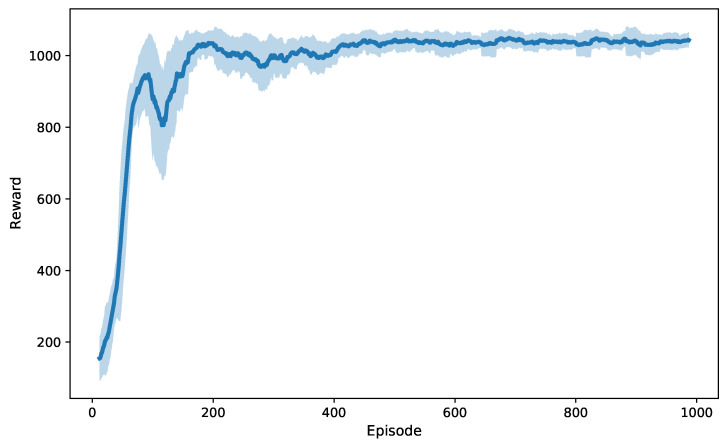
Training reward curve of SAC algorithm.

Figure 3 illustrates the evolution of the system reward during SAC algorithm training as a function of episodes. It can be observed that in the early stages of training, the reward curve exhibited significant fluctuations, reflecting the typical characteristics of random exploration and policy updates. As training progressed, the system reward gradually increased and the fluctuations decreased, eventually reaching a steady state, which indicated that the SAC agent’s policy gradually converged and stabilized around the optimal or near-optimal solution.

**Figure 4:**
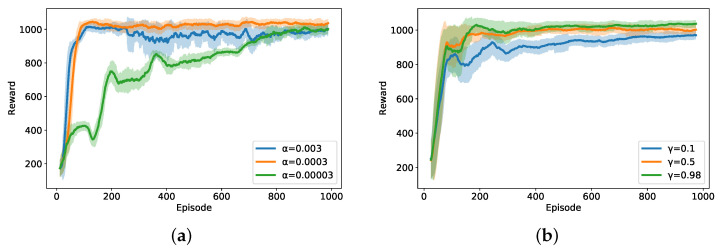
Convergence characteristics of the SAC algorithm with different hyperparameters: (**a**) impact of learning rate; (**b**) impact of discount factor.

Figure 4 presents the convergence characteristics of the SAC algorithm under different hyperparameter configurations to demonstrate the robustness of our hybrid optimization framework against hyperparameter variations. The results reveal that the algorithm achieved relatively stable performance across various learning rates and discount factors, which validates the effectiveness of our architectural design, which combines closed-form power allocation solutions with efficient user scheduling to constrain the SAC search space.

**Figure 5:**
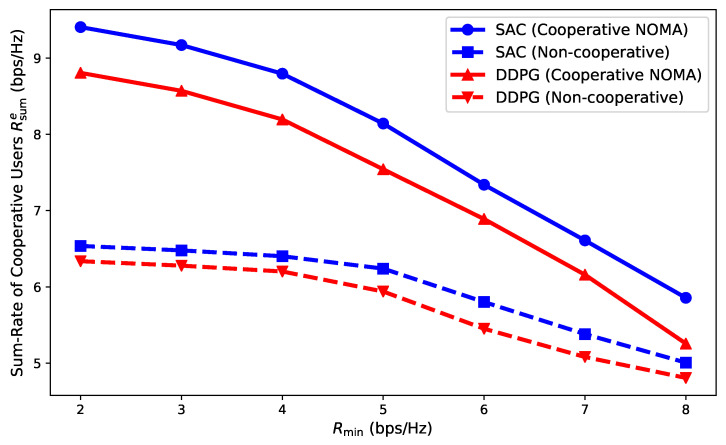
Impact of minimum rate requirement on cooperative user performance.

Figure 5 compares cooperative user rate performance under different minimum rate requirements for cooperative NOMA and non-cooperative schemes, each trained with SAC or DDPG. The cooperative user rate decreased as minimum rate requirements increased. The proposed cooperative NOMA scheme combined with SAC consistently achieved the highest cooperative user rates, outperforming all other schemes. The DDPG-based cooperative scheme performed better than the corresponding non-cooperative scheme but was outperformed by SAC-based solutions. These results demonstrate the superiority of integrating cooperative transmission and SAC learning, especially under stringent rate constraints.

**Figure 6:**
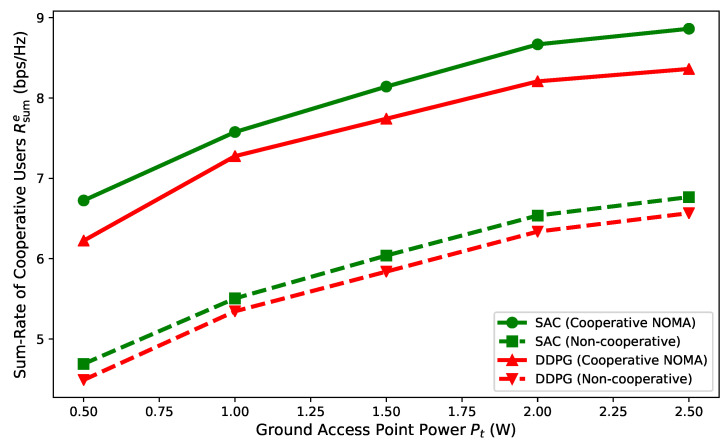
Impact of ground access point transmit power on cooperative user rate.

Figure 6 shows the effect of increasing GAP transmit power on cooperative user rates. As the GAP power increased, cooperative user rates rose, with the cooperative NOMA scheme demonstrating substantial gains over the non-cooperative scheme. This improvement was attributed to the proposed scheme’s effective interference management via UAV–GAP cooperative transmission, which mitigated interference from the GAP- to UAV-served users.

**Figure 7:**
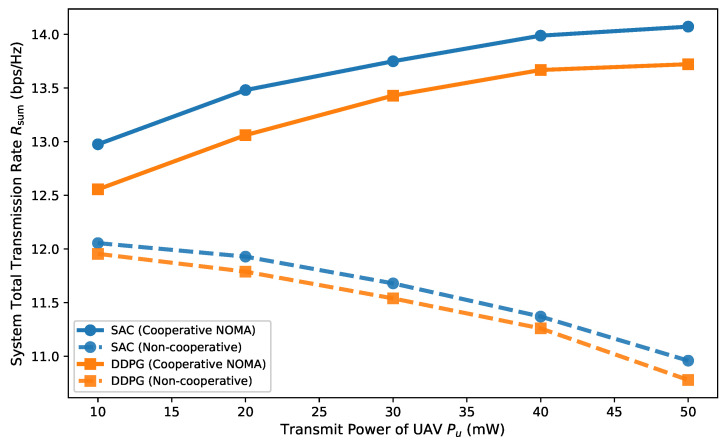
System sum rate with varying UAV transmit power.

Figure 7 illustrates the overall system sum rate as a function of UAV transmit power, with the GAP power fixed at 1.5 W and the minimum rate requirement at 5 bps/Hz. For the cooperative NOMA scheme, while increasing UAV power improved the sum rate, the growth rate slowed as power continued to rise. This phenomenon occurred because although cooperative NOMA boosted cooperative user rates, higher UAV power also intensified interference for non-cooperative users within NOMA groups, constraining overall throughput gains. In contrast, for the non-cooperative scheme, the system sum rate decreased as UAV transmit power increased since the UAV acted purely as an interference source without providing beneficial cooperative transmission to users served by the GAP.

**Figure 8:**
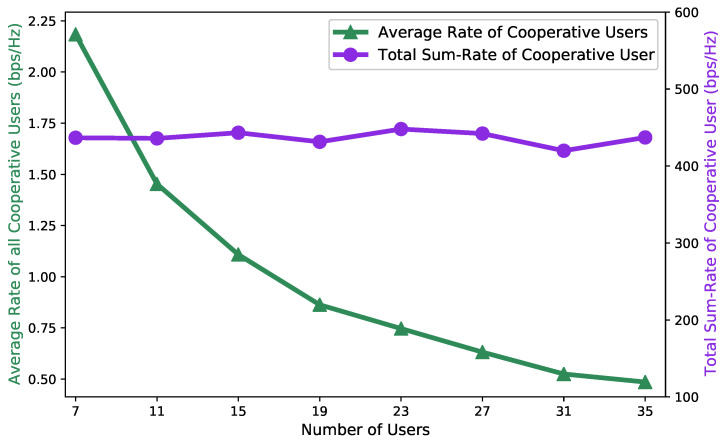
System performance with varying numbers of users.

Figure 8 demonstrates system performance with varying numbers of users. The simulation results show a decreasing trend in average cooperative user rate as the number of users increased, which was expected due to the fundamental resource-sharing nature of the system. Specifically, while the SAC-based algorithm maintained robust resource allocation strategies that kept the total system throughput relatively stable, the increasing number of cooperative users led to more intensive resource competition. Consequently, when the total achievable rate was divided among a larger number of cooperative users, the per-user average rate naturally decreased. This trend validates the algorithm’s ability to maintain system-wide performance stability while fairly distributing resources among an expanding user population.

**Figure 9:**
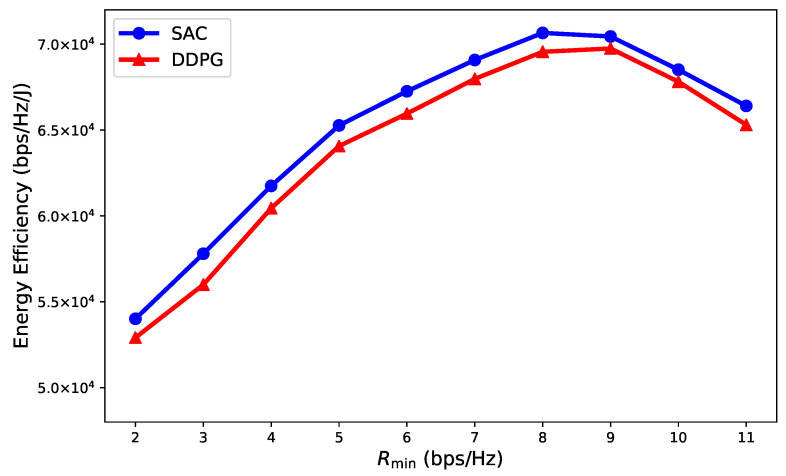
Energy efficiency performance for UAV-GAP cooperative NOMA systems.

In Figure 9, we evaluate the energy efficiency (EE) performance of the proposed SAC and DDPG algorithms, considering both transmit power and propulsion power of the UAV. We set $P_{t}=1$ W and $P_{u}=30$ mW. The EE of the UAV-GAP cooperative NOMA systems could be obtained as $EE=\frac{B}{N}\sum_{n=1}^{N}\frac{\sum_{i\in L_{c}}S_{i,n}R_{i,n}+\sum_{l\in L_{e}}S_{l,n}R_{l,n}}{P\left(v_{n}\right)+P_{u}+\sum_{j\in {≍}_{n}}P_{j,n}}.$ Here, $P(v_{n})$ represents the propulsion power, which can be regarded as a function of the UAV’s flight speed $v_{n}$. As can be seen, there was a trade-off between system transmission rate and total power consumption, and the proposed SAC-based cooperative algorithm achieved superior EE performance compared to DDPG.

**Figure 10:**
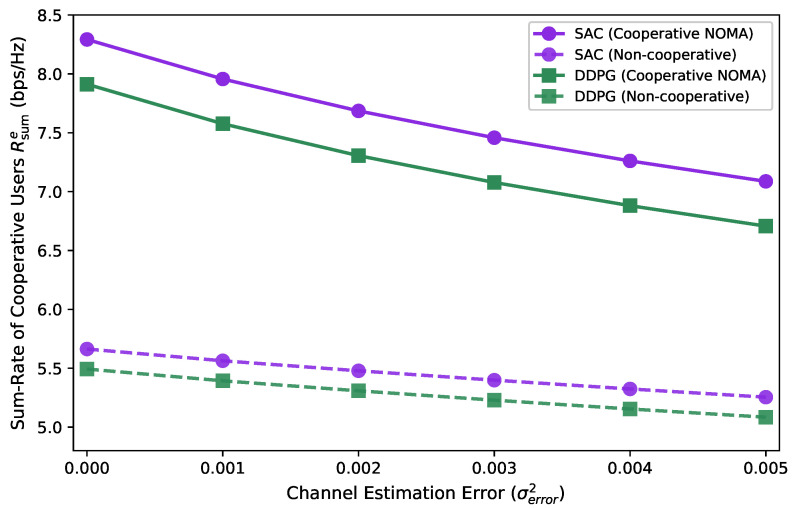
Effect of channel estimation error on cooperative user rate.

Figure 10 compares edge user rates under varying channel estimation errors across cooperative and non-cooperative modes trained with SAC and DDPG. Edge rates degraded as estimation error increased. The SAC-based cooperative scheme consistently achieved the highest rates, outperforming other schemes. Non-cooperative approaches suffered from a lack of coordination, resulting in lower performance and greater sensitivity to estimation errors. The results highlight that integrating cooperative transmission with high-performance learning algorithms improves robustness against channel uncertainties.

**Figure 11:**
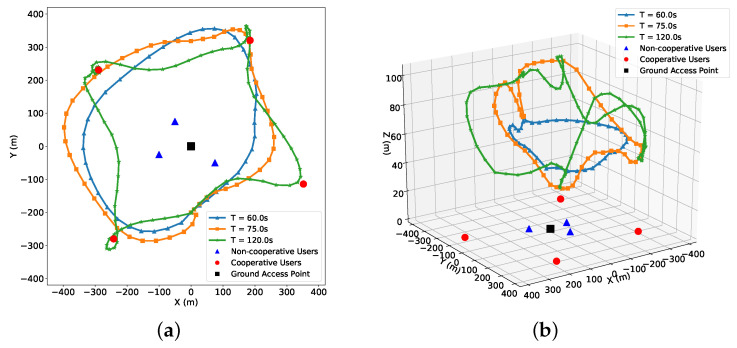
UAV trajectory illustration with user locations. (**a**) UAV trajectory top-down view, (**b**) UAV trajectory 3D view.

Figure 11 illustrates the UAV trajectory in both 2D and 3D views under varying flight durations *T* = 60 s, 75 s, and 120 s, with a minimum edge user rate requirement $R_{\min}=5$ bps/Hz. All ground users were located at zero altitude. For a short flight time (*T* = 60 s), the UAV trajectory was compact and flight distance was limited due to constrained time and space, forcing the UAV to serve all users within a smaller region. As the flight duration was extended (*T* = 75 s), the UAV exploited its 3D mobility more flexibly, approaching users more closely and adjusting the altitude to optimize channel conditions. With sufficient flight time (*T* = 120 s), the UAV executed more diverse trajectories with wider coverage and dynamic service adaptation, fully leveraging its maneuverability to satisfy communication demands.

## 6. Conclusions

This paper presented an innovative framework for intelligent resource allocation and 3D trajectory optimization in UAV-GAP cooperative NOMA systems based on the SAC deep reinforcement learning algorithm. By integrating power allocation and user scheduling into the reinforcement learning decision-making process and utilizing the SAC algorithm to handle continuous action spaces, we successfully achieved adaptive optimization of UAV trajectories, thereby significantly improving system performance. Experimental results demonstrated that compared to traditional non-cooperative and DDPG algorithms, our proposed scheme exhibited superior performance in enhancing overall system throughput, improving spectral efficiency, effectively managing interference, and guaranteeing the quality of service for cooperative users. Particularly in dynamic and complex industrial IoT scenarios, this framework showed stronger adaptability and robustness, capable of effectively addressing changes in channel conditions and the impact of errors. Future research directions should include the further exploration of multi-UAV cooperation and integrated sensing and communication and the consideration of UAV energy efficiency constraints to meet more complex Industrial application requirements.

## Figures and Tables

**Table 1:** Simulation parameters.

Notation	Description	Value
*B*	System bandwidth	1 MHz
$P_{t}$	Ground base station transmit power	1.5 W
$P_{u}$	UAV transmit power	40 mW
$K_{c}$	Number of non-cooperative users	3
$K_{e}$	Number of cooperative users	4
*H*	UAV initial flight altitude	70 m
$v_{\max}$	UAV maximum horizontal speed	50 m/s
$v_{\max,z}$	UAV maximum vertical speed	20 m/s
*N*	Number of time slots	80
D	UAV flight area	[−400,400]×[−400,400]×[50,100] m
$R_{\min}$	Minimum rate requirement per user	4 bits/s/Hz
$\sigma^{2}$	Noise power spectral density	−110 dBm
*a*, *b*	Urban LoS channel parameters	4.88, 0.43
α	Path loss exponent	3
$L_{c}$, $L_{e}$	Max scheduling counts for center and edge users	27, 23

**Table 2:** SAC algorithm training parameters.

Symbol	Parameter	Value
$\alpha_{a}$	Actor learning rate	0.0003
$\alpha_{c}$	Critic learning rate	0.0003
γ	Discount factor	0.98
|B|	Replay buffer size	$10^{6}$
*B*	Batch size	64
τ	Target network update rate	0.005
*E*	Episodes	1000
*N*	Time slots per episode	80
*L*	Neural network layers	3
*H*	Hidden units per layer	128

## Data Availability

The data presented in this study are available upon request from the corresponding author. The data are not publicly available due to the copyright.
